# Enhanced Hemodynamic and Clinical Response to αCGRP in Migraine Patients—A TCD Study

**DOI:** 10.3389/fneur.2021.638903

**Published:** 2021-01-28

**Authors:** Darja Visočnik, Marjan Zaletel, Bojana Žvan, Matija Zupan

**Affiliations:** Department of Neurology, University Medical Center Ljubljana, University of Ljubljana, Ljubljana, Slovenia

**Keywords:** migraine, TCD, CGRP-induced headache, nervous system sensitization, middle cerebral artery

## Abstract

**Introduction:** Sensitisation of the nervous system in a patient with migraine is supposed to be associated with calcitonin gene-related peptide (CGRP) activity. Therefore, the vascular response to human αCGRP (hαCGRP) could be a surrogate marker for the sensitization. We hypothesize that vascular response to hαCGRP is augmented in a patient with migraine.

**Methods:** Twenty healthy subjects and 20 patients with migraine participated in our study. TCD was used to monitor mean arterial velocity in the middle cerebral artery (vm MCA). Simultaneously, end-tidal CO_2_ (Et-CO_2_), mean arterial pressure (MAP), and heart rate (HR) were measured. The reconstruction of the signals was made for basal conditions, during and after CGRP infusion which were compared using statistics.

**Results:** In both groups, we found significant decrease between measurement points of vm MCA and Et-CO_2_ during and after hαCGRP infusion. MAP did not show significant trends during the infusion, but it was significantly increased after the infusion in migraine patients only. Responses to hαCGRP, defined as differences between two measurement points, were significantly higher for vm MCA and Et-CO_2_ in patients with migraine. A significant difference between groups was found in MAP response. Significant relationships were found between migraine and vm MCA, Et-CO_2_, and MAP.

**Conclusion:** In patients with migraine, vm MCA responses to hαCGRP are significantly higher and are associated with CGRP-induced headache which indicates that patients with migraine are more prone to sensitization.

## Introduction

Migraine is recognized as an episodic or chronic state of nervous system sensitization involving a CGRP-dependent process (1). It is well-known that CGRP induces vasodilatation and enhances neuronal transmission (2). Therefore, the level of brain sensitization could be reflected in CGRP induced cerebral hemodynamic changes detected by transcranial Doppler sonography (TCD). The vascular biomarker CGRP could be a useful clinical tool to assess sensitization interictally and to determine treatment responses for monoclonal antibodies.

Until now, only a few studies have investigated exogenous hαCGRP response of cerebral circulation. In two previous studies, they monitored the vascular responses in healthy subjects using TCD (3) and magnetic resonance angiography (4). The results regarding the effects on MCA were conflicting. In the first study they reported vasodilatation of MCA, but in the second study no noticeable changes in MCA were observed (5). Lassen et al. performed the study in patients with migraine using TCD and 133-Xe SPECT. They found decreased mean arterial velocity in MCA (vm MCA) during hαCGRP infusion indicating a dilation of the MCA. The same group found that the increase in plasma CGRP caused headache in patients with migraine (6). Therefore, available studies did not compare the responses to hαCGRP between healthy subjects and patients with migraine and systematically study them.

Several studies on cerebrovascular reactivity in migraine observed consistently increased responses to visual stimuli, but inconsistent responses to systemic stimuli such as hypo- or hypercapnia (7). This suggested the importance of the central mechanism and evidence of a hyper-responsive state due to sensitization of nervous system in migraine reflected in cerebral circulation. Thus, we expected an increase of the MCA vasodilatory response to hαCGRP in migraine along with the occurrence of the CGRP-induced headache. Additionally, we anticipated a positive association between vm MCA and CGRP-induced headache.

## Methods

In our study we included 20 healthy subjects (nine females aged 37.0 ± 2.8 years, 11 males aged 41.8 ± 7.6 years), which represented the control group, and 20 patients with migraine (15 females aged 41.9 ± 9.9 years, five males aged 38.2 ± 9.2 years), which represented the study group. We did not find statistically significant differences in sex (*p* = 0.105) and in age (*p* = 0.066) between the groups. The inclusion criteria for the group of healthy participants were age 18 years or more, without migraine history, normal somatic and neurological status, and normal carotid and vertebral arteries ultrasound examination. The exclusion criteria for the group of healthy subjects were pregnancy and breastfeeding. The inclusion criteria for the group of migraineurs were age 18 years or more, history of migraine with or without aura in accordance to the ICHD-3 criteria of the International Headache Society, normal somatic and neurological status and without any hemodynamically significant atherosclerotic processes of the carotid and vertebral arteries. The exclusion criteria for this group were current or previous cerebrovascular, renal or liver diseases, uncontrolled hypertension, pregnancy and breast-feeding. The subjects with migraine were free of migraine headache at least 24 h before the experiment.

The participants of both groups were free of tobacco, coffee, tea or any other food or beverages containing caffeine for at least 12 h before the start of the measurements.

All participants were given a written explanation concerning the experimental procedure and were informed that they were free to withdraw from the study at any time. They all gave written consent to participate in the study. The study was approved by the National Medical Ethics Committee of the Republic of Slovenia.

Before the beginning of the experiment, color-coded duplex sonography of the carotid and vertebral arteries was performed using the standard procedure. The experiments took place at 9:00 am in a quiet room under constant conditions. During the experiment, the participants were resting in a supine position. An intravenous cannula was placed in the left cubital vein for hαCGRP infusion. The experiment consisted of a 10 min baseline period, a 20 min period during which an intravenous infusion of hαCGRP 1.5 mcg/min (Calbiochem, Merck4Biosciences, Darmstadt, Germany) was given and a 10 min period after the end of the application of hαCGRP. The hαCGRP dose was chosen based on the finding of previous studies so as not to induce pronounced hypotension (3).

Transcranial Doppler (TCD) sonography with 2 MHz ultrasound probes was applied to measure mean flow velocity (vm) in left MCA through the left transtemporal acoustic window. The signals of the MCA were defined according to the direction of the blood flow and typical depth of the signal. A mechanical probe holder was used to ensure a constant probe position. During the entire experiment mean blood pressure (MAP) and heart rate (HR) were continuously measured using non-invasive plethysmography (Colin 7000, 12 Komaki-City, Japan). The Et-CO_2_ was measured by a ventilation mask and an infrared capnograph (Capnograph, Model 9004, Smith Medical, USA) using the standard protocol. The capnograph was connected to a computer. Et-CO_2_ signals were recorded on the same time scale as other variables.

TCD Multi-Dop X4 software (DWL, Sipplingen, Germany) was used to define mean values of vm MCA, MAP, HR, and Et-CO_2_ during 5 min intervals: one interval during the baseline period (5–10 min of the experiment-measurement point 1), two intervals during the hαCGRP infusion (15–20 min-point 2 and 25–30 min-point 3 of the experiment), and one interval after the end of hαCGRP infusion (35–40 min of the experiment-point 4). The mean vm MCA was calculated for each 5-min interval using the following equation: vm = ∫ vdt/ (t0 – t5).The mean values of other variables (MAP, HR, and Et-CO_2_) were also calculated for the same time intervals as vm MCA using TCD software.

We determined responses to hαCGRP as the differences between measuring points for vm MCA, Et-CO_2_, MAP, and HR, respectively. The response 1 represented the difference between points 1 and 2, the response 2 between points 1 and 3 and the response 3 between the measurement points 1 and 4.

The incidence of CGRP-induced headaches was recorded within 1 h of the experiment (immediate CGRP-induced headache) and within the next 12 h after the experiment (delayed CGRP-induced headache).

The number of the subjects enrolled in the study was driven from previous studies (3–5). For statistical analysis, SPSS version 21 was used. Paired *t*-test was used to test the significance of differences between two measurement points in the same group and ANOVA for responses between the groups. Linear and logistic regressions were used to test the correlations between the variables. Chi-square was used to test the difference among the frequencies. A *p*-value < 0.05 was considered to represent a statistically significant difference or relationship. We used a multivariate model, which includes migraine as the covariate to test the relationship between the responses of all previously mentioned parameters.

## Results

First, we analyzed the occurrence of the immediate and delayed CGRP-induced headache in healthy and migraine patients. An immediate headache was found in seven migraine patients (35%) and in three healthy participants (15%) with a statistically significant difference between the groups (*p* = 0.010) while delayed headaches appeared in 13 migraine patients (65%) and one healthy person (5%) (*p* < 0.01). Migraine was significantly related to immediate CGRP-induced headaches (OR = 3.05, 95%CI 1.25–7.39; *p* = 0.014) and delayed CGRP-induced headaches (OR = 35.26; 95%CI 9.84–126.45; *p* < 0.001) as well.

**Figure 1:**
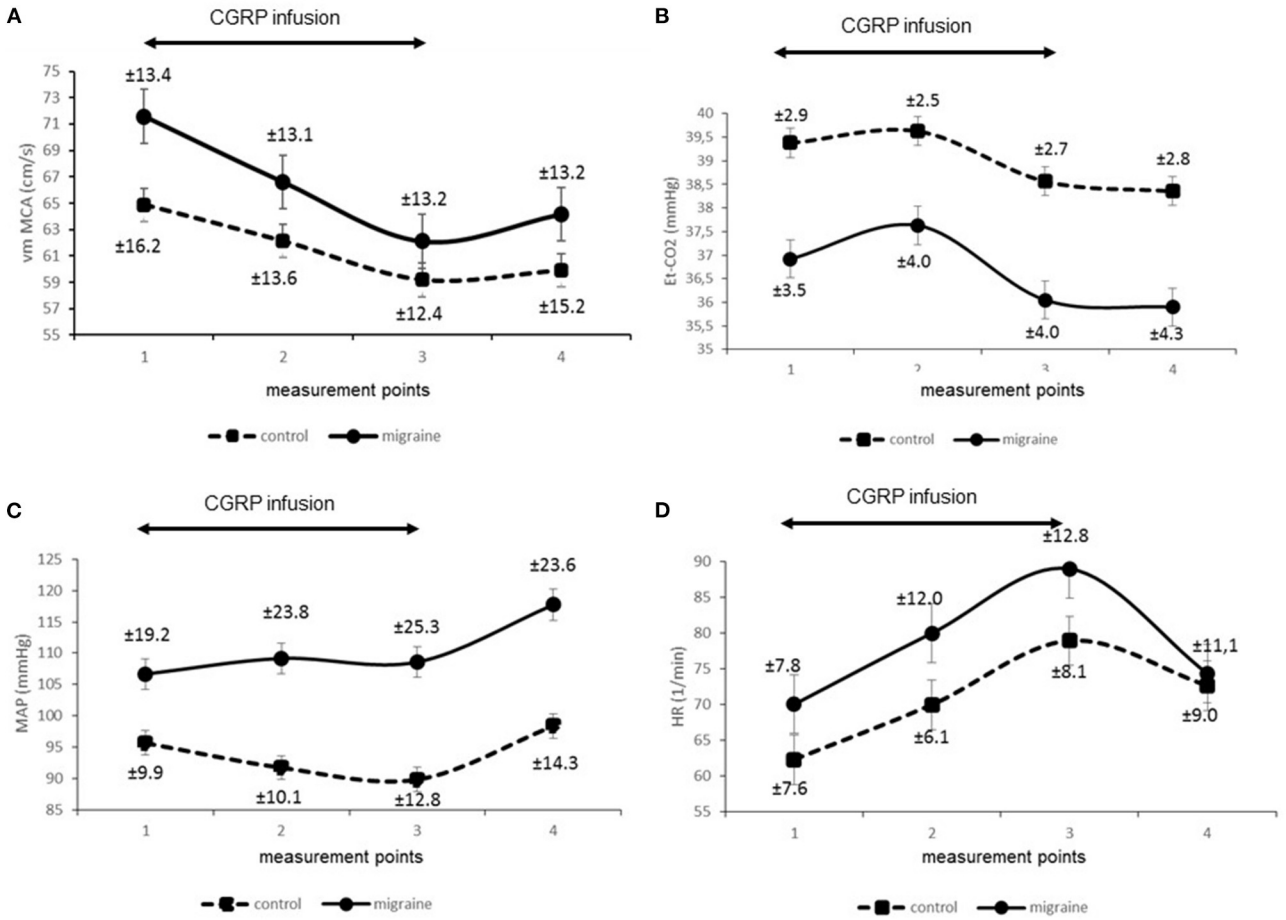
**(A-D)** Time course of physiologic signals during and after hαCGRP infusion.

Secondly, we compared signals of parameters obtained from the migraine patients and compared them to the control group. Figure 1 presents reconstructed signals for vm MCA (Figure 1A), Et-CO_2_ (Figure 1B), MAP (Figure 1C), and HR (Figure 1D). The differences between two measuring points for both groups were tested using paired *t*-test and are represented in Table 1.

**Table 1:** Significance of differences between measurement points.

**Healthy subjects**	**Point 1–2**	**Point 1–3**	**Point 1–4**
vm MCA	*p* < 0.001	*p* < 0.001	*p* < 0.001
Et-CO_2_	*p* = 0.376	*p* = 0.023	*p* = 0.066
MAP	*p* = 0.062	*p* = 0.027	*p* = 0.119
HR	*p* < 0.001	*p* < 0.001	*p* < 0.001
**Migraine patients**			
vm MCA	*p* < 0.001	*p* < 0.001	*p* < 0.001
Et-CO_2_	*p* = 0.018	*p* < 0.001	*p* = 0.001
MAP	*p* = 0.226	*p* = 0.557	*p* = 0.001
HR	*p* < 0.001	*p* < 0.001	*p* < 0.001

**Figure 2:**
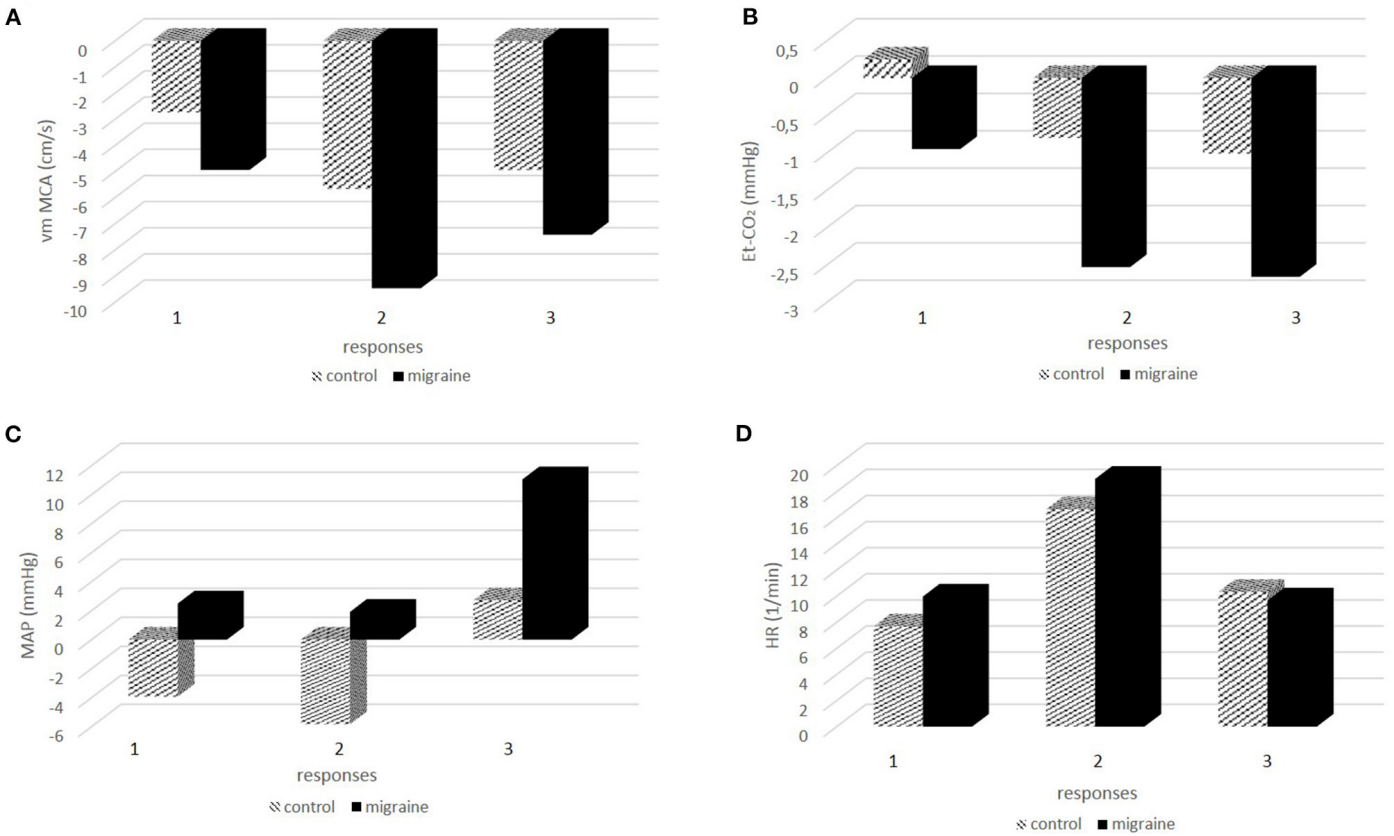
**(A-D)** The responses of physiologic signals during and after hαCGRP infusion between the control group and patients with migraine.

In the next step, we tested the responses between the measuring points 1–2 (response 1), 1–3 (response 2), and 1–4 (response 3) between the control group and patients with migraine. The results are presented in Figure 2. Figure 2A presents responses for vm MCA, Figure 2B for Et-CO_2_, Figure 2C for MAP, and Figure 2D for HR. ANOVA showed significant differences in vm MCA response 2 (*p* = 0.017) but not in MCA response 1 (*p* = 0.085) and MCA response 3 (*p* = 0.103), although the probabilities were borderline. The response of Et-CO_2_ was significant for response 1 (*p* = 0.012) and response 2 (*p* = 0.007) but borderline for response 3 (*p* = 0.057). MAP showed significant differences in response 1 (*p* = 0.034) and response 3 (*p* = 0.018) and borderline in response 2 (*p* = 0.086). HR between the groups was not significantly different for any response; response 1 (*p* = 0.375), response 2 (*p* = 0.520), and response 3 (*p* = 0.741).

Since the Et-CO_2_ response could directly affect vasodilatation and the increase of CBF, we tested the relationship between Et-CO_2_ and vm MCA responses. Linear regression showed significant associations between the variables (B = 0.22; beta = 0.48; *p* < 0.001).The relationship between vm MCA, Et-CO_2_, MAP, HR, and migraine was tested using logistic regression. We found significant associations between migraine and vm MCA (OR = 1.15; 95%CI 1.05–1.25; *p* = 0.003), Et-CO_2_ (OR = 1.45; 95%CI 1.17–1.80; *p* = 0.001), MAP (OR = 0.93; 95%CI 0.90–0.97; *p* = 0.001) but not for HR (OR = 0.98; 95%CI 0.94–1.03; *p* = 0.986).

Finally, we tested the relationships between CGRP-induced headache and physiologic variables using logistic regression. The CGRP-induced headache represented the dependent and vm MCA presented the independent variable. We predicted that CGRP-induced headache refereed to vasodilatatory response and vm MCA changes. Logistic regression showed the association between immediate CGRP-induced headache and vm MCA response (OR = 1.16; 95%CI 1.06–1.27; *p* = 0.002) but not between delayed headache and vm MCA response (OR = 1.05; 95%CI 0.97–1.14; *p* = 0.170). Interestingly, we found a statistically significant relationship between Et-CO_2_ response and delayed CGRP-induced headache (OR = 1.29; 95%CI 1.07–1.55; *p* = 0.006) but not immediate CGRP-induced headache (OR = 1.06; 95%CI 0.88–1.26; *p* = 0.511).

## Discussion

The main finding of our study was a uniform decrease of vm MCA in both healthy controls and in migraine patients. This observation is in accordance with the results of previous studies obtained in healthy subjects and in migraine patients, although in healthy subjects the results were ambiguous (3, 5). Therefore, the contribution of our study is clarification of the phenomenon in the healthy control group.

Furthermore, we found an enhanced response of vm MCA in migraine and a positive relationship between vm MCA responses and migraine. According to the current model of migraine, these results can be attributed to a pronounced vasodilatory response of MCA to CGRP in migraine. This finding is supported by the results of visually evoked flow studies, which found higher cerebral vascular responses to visual stimulation in patients with migraine compared to healthy controls (8, 9).

It is broadly accepted that migraine is neurogenic in origin while vascular changes reflect the state of the nervous system through the mechanism of neurovascular coupling. In our study, intravascularly applied hαCGRP induced vasodilatation of MCA in controls as well as in migraineurs. The putative target of hαCGRP is a trigeminal ganglion lacking blood brain barrier or peripheral meningeal tissue including mast cells (10). In the meninges, CGRP likely contributes to neurogenic inflammation by triggering the release of neuron sensitizing agents from mast cells, which in turn leads to increased vasodilation in the dura (11). Consequently, activation of the trigeminovascular reflex (TVR) induces vasodilatation and in some cases may produce neurogenic inflammation reflecting in the CGRP headaches detected in our study. In addition, we observed CGRP-induced headaches among subjects without migraine but in the migraine group, not all subjects reported CGRP headaches. Thus, we assume that resting state TVR was different between the control group and the migraine group and even among individuals in both groups. Apparently, hαCGRP induced vascular changes in migraine patients are reflected in the hypersensitivity state of the central nervous system in general, which is not specific for migraine patients but it could occur more frequently among them. The hypersensitivity state can be found in other pathological states without migraine such as widespread pain syndrome (12).

An interesting finding of our study was a more pronounced decrease of Et-CO_2_ in the migraine group. Thus, hαCGRP-induced decrease of Et-CO_2_ was a consistent response in the control group as well as in migraine patients. This is relatively surprising since it has been reported in a previous study (5), but it has not attracted attention in terms of physiological significance. Our methods enabled us to compare the signals obtained on the same time scale. Accordingly, we observed that Et-CO_2_ follows vm MCA changes and we found a significant linear positive relationship between both signals. In addition, we found an important relationship between migraine and Et-CO_2_. We could interpret the findings as physiologically related phenomena.

According to recent knowledge and concepts, intravascular CGRP induces vasodilatation in proximal and distal vascular segments of cerebral blood vessels, changes the resistance and increases cerebral blood flow (CBF) (13). It is accepted that cerebral circulation is not simply a reflection of the systemic one. In the principle, the human cerebral circulation is divided in the proximal and distal segments regarding to the overall cerebral resistance, which is accordingly consisted of upstream and downstream resistance. Both segments are important for governing microvascular pressure with implications for regional flow distribution of parallel vessels. Accordingly, resistance of cerebral blood vessels is organized in series of upstream and downstream small vessels. The upstream large vessels, including MCA, contribute about 50% of the overall cerebral resistance. The downstream small vessels such as intra-parenchymal arterioles, contribute the rest of 50% of the overall cerebral resistance. Both segments act synchronously to regulate CBF (13). Our study indicates that CGRP-induced vasodilatation of intracranial arteries includes proximal segments of the cerebral arteries, which in turn increases CBF. The following raises concern over the safety of the CGRP pathway inhibiting therapy. Nevertheless, recent studies have shown that global cerebral vasodilatory reserve remains preserved and endothelial function does not seem to change after treatment with anti-CGRP monoclonal antibodies (14). Therefore, it seems that CGRP acts mainly on proximal segments of cerebral arteries and affect global cerebral vasodilatory capacity. This supports the thesis that CGRP-induced vasodilation of large cerebral vessels is just an epiphenomenon of the trigeminovascular complex sensitization.

Since CBF under the constant conditions is an imperative of cerebral hemodynamics, the Et-CO_2_ could be a putative factor counteracting the CGRP vasodilatory effect. This is a well-known phenomenon named vasomotor reactivity to hyper/hypocapnia, and it is one of several mechanisms to ensure a constant level of CBF (15), which could explain why the variables were associated in our analysis. The reason for a more pronounced response of Et-CO_2_ in migraine could be attributed to the hypersensitivity neurological state that is associated with migraine. In our study we found that Et-CO_2_ responses were associated with delayed CGRP headaches but not to immediate ones. On the contrary, vm MCA was associated with immediate CGRP headaches. It seems that CGRP itself, with immediate vasodilatation, is responsible for immediate CGRP-induced headaches, while delayed headaches are associated with the compensatory processes in cerebral circulation. It is known that in migraine patients elevated CGRP levels are normalized after sumatriptan administration (16), which could explain the highest effectiveness of triptans in the early phase of migraine headaches.

In addition, we monitored systemic variables in both groups and found significant differences in some MAP responses but not in HR responses between the groups, as well as differences in baseline hemodynamic parameters. We believe the latter not to be important to our study since we focused on the changes of hemodynamic parameters during CGRP stimulation and not on their absolute baseline values. In two previous studies, a decrease of MAP was found in migraine patients (6) but not in healthy volunteers (3). A more remote study reported a decrease of arterial pressure even in the healthy control group (17). In our study we detected differences in MAP responses in the migraine group after the administration of the hαCGRP, but not during the infusion. Accordingly, we can deduce that hαCGRP in a dose of 1.5 mcg/min does not have direct significant effects on MAP in migraine. This is in accordance with the finding that blocking CGRP does not affect systemic blood pressure in healthy volunteers (18).

The distinction in systemic effects might be linked to the endothelial dysfunction in migraine which is still a debatable issue, but could explain the association between migraine and the risk of vascular disease (19).

Nevertheless, in the present study we found a significant positive chronotropic effect of CGRP, which has already been recognized in previous studies (20).

The main limitation of our study was the lack of data on blood CGRP concentrations in the study subjects during the experiment. In addition, we did not test placebo effects on CGRP headaches. In our study we did not explore the difference in hemodynamic responses between migraine with and without aura. However, previous studies demonstrated that patients with migraine with aura might have excessive response to several stimuli (20, 21). Further studies are needed to investigate the CGRP induced differences between migraine with aura and migraine without aura (21, 22).

## Conclusion

In conclusion, hαCGRP could induce vasodilatation of MCA probably *via* activation of TVR. Decrease of Et-CO_2_ might reflect a compensatory decrease in arterial partial pressure of CO_2_, which underlies the normalization of CBF during CGRP activity. It seems that immediate CGRP-induced headaches are caused by the direct CGRP effects on neurovascular structures, while delayed CGRP-induced headaches are an indirect CGRP phenomenon due to compensatory processes. CGRP had chronotropic effects in both the migraine patients and the control group, while systemic hemodynamic was not importantly affected in either group.

## Data Availability Statement

The raw data supporting the conclusions of this article will be made available by the authors, without undue reservation.

## Ethics Statement

The studies involving human participants were reviewed and approved by National Medical Ethics Committee of Republic of Slovenia. The patients/participants provided their written informed consent to participate in this study.

## Author Contributions

All authors made a substantial intellectual contribution to the study. DV, BŽ, and MZ made conception and design. MZ made analysis and interpretation of the data. All authors approved the final version of the article.

## Conflict of Interest

The authors declare that the research was conducted in the absence of any commercial or financial relationships that could be construed as a potential conflict of interest.
